# MHC Class II–Alpha Chain Knockout Mice Support Increased Viral Replication That Is Independent of Their Lack of MHC Class II Cell Surface Expression and Associated Immune Function Deficiencies

**DOI:** 10.1371/journal.pone.0068458

**Published:** 2013-06-28

**Authors:** Mohammed Alsharifi, Aulikki Koskinen, Danushka K. Wijesundara, Jayaram Bettadapura, Arno Müllbacher

**Affiliations:** Department of Immunology, The John Curtin School of Medical Research, Australian National University, Canberra, Australian Capital Territory, Australia; Wayne State University, United States of America

## Abstract

MHCII molecules are heterodimeric cell surface proteins composed of an α and β chain. These molecules are almost exclusively expressed on thymic epithelium and antigen presenting cells (APCs) and play a central role in the development and function of CD4 T cells. Various MHC-II knockout mice have been generated including MHC-IIAα^-/-^ (I-Aα^-/-^), MHC-IIAβ^-/-^ (I-β^-/-^) and the double knockout (I-Aαxβ^-/-^). Here we report a very striking observation, namely that alphaviruses including the avirulent strain of Semliki Forest virus (aSFV), which causes asymptomatic infection in wild-type C57BL6/J (B6) mice, causes a very acute and lethal infection in I-Aα^-/-^, but not in I-β^-/-^ or I-Aαxβ^-/-^, mice. This susceptibility to aSFV is associated with high virus titres in muscle, spleen, liver, and brain compared to B6 mice. In addition, I-Aα^-/-^ mice show intact IFN-I responses in terms of IFN-I serum levels and IFN-I receptor expression and function. Radiation bone marrow chimeras of B6 mice reconstituted with I-Aα^-/-^ bone marrow expressed B6 phenotype, whereas radiation chimeras of I-Aα^-/-^ mice reconstituted with B6 bone marrow expressed the phenotype of high viral susceptibility. Virus replication experiments both in vivo and in vitro showed enhanced virus growth in tissues and cell cultures derived form I-Aα^-/-^ compared to B6 mice. This enhanced virus replication is evident for other alpha-, flavi- and poxviruses and may be of great benefit to producers of viral vaccines. In conclusion, I-Aα^-/-^ mice exhibit a striking susceptibility to virus infections independent of their defective MHC-II expression. Detailed genetic analysis will be carried out to characterise the underlining genetic defects responsible for the observed phenomenon.

## Introduction

The interplay between the innate and the adaptive arms of the immune system is essential for the establishment of protective immunity against harmful microbial infections. This protective immunity involves differential recognition of infectious non-self antigens. CD4^+^T cells, part of the adaptive immune response, play a major role in orchestrating both humoral and cell-mediated immunity. Importantly, helper and regulatory functions of CD4^+^ T cells involve antigen recognition through their TCRs, which recognize processed peptide antigens presented by antigen presenting cells (APCs) in the context of major histocompatibility complex class II (MHC-II) molecules. Therefore, MHC-II molecules play a central role in the development and function of the immune system. Expression of MHC-II genes is tightly regulated and mostly restricted to thymic epithelium [1], [2] and APCs [3], [4]. MHC-II molecules are heterodimeric proteins and there are two isotypic forms (I-A and I-E) expressed in mice. Each isotypic form is composed of an α and β chain (Aα:Aβ and Eα:Eβ, respectively). Thus, any strategy to generate MHC-II-deficient (MHC-II^-/-^) mice requires the elimination of both I-A and I-E expression. It is fortunate that H-2^b^ mice (such as C57BL6/J mice) have a deletion in their Eα gene, resulting in their inability to produce Eα proteins [5]. As the pairing of both chains is required for stable cell surface expression, H-2^b^ mice lack I-E expression. It follows that disruption of either Aα or Aβ gene locus does prevent the cell surface expression of I-A molecules. For the purposes of understanding MHC-II function in mice, several H-2^b^ strains completely defective in MHC-II expression have been generated by targeted disruption of the Aβ gene (I-Aβ^-/-^) [1], [6], the Aα gene (I-Aα^-/-^) [7] and by deletion of both the Aα and Aβ locus (I-Aαxβ^-/-^) [8]. Using one or other of these ko strains, the function of MHC-II molecules in immune responses has been investigated over the past twenty years. Most studies have used I-Aβ^-/-^ mice to examine the role of MHC-II in the response to virus infection, such as influenza [9]–[11], LCM [12]–[14], Theiler [15], [16], Vaccinia [17], and Sendai [18]. I-Aα^-/-^ mice have only been referred to in two research articles investigating ectromelia (ECT) virus (orthopoxvirus) [19] and Lassa virus infection [20]. Despite defective Ab-isotype switching, these studies did not report marked increases in susceptibility to viral infections. Here we report that avirulent SFV (aSFV), which only causes asymptomatic infection in adult wild-type C57BL6/J (B6) mice, caused a very acute and lethal infection in I-Aα^-/-^ mice. We found this susceptibility to be unrelated to the lack of functional MHC-II expression, but associated with increased virus replications in various tissues. Such increased virus susceptibility appears to be associated with viral infections in general.

## Results and Discussion

**Figure 1:**
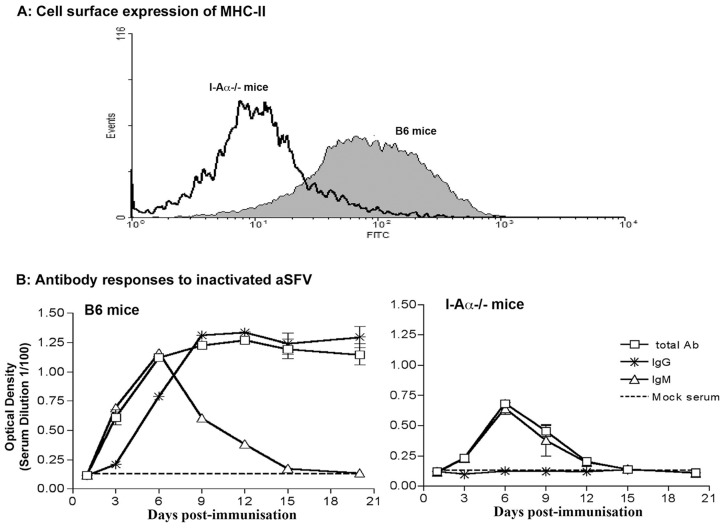
Defective MHC-II expression and Ab response in I-Aα^-/-^ mice. A) Cell surface expression of MHC-II on CD19^+^ cells from B6 and I-Aα^-/-^ mice. B) B6 and I-Aα^-/-^ mice were immunised with gamma-irradiated SFV and sera analysed for Ab responses and isotype switching.

The absence of functional MHC-II molecule expression in I-Aα^-/-^ mice was demonstrated by a lack of cell surface expression of MHC-II and antibody isotype response to inactivated viruses (figure 1). Splenocytes from B6 and I-Aα^-/-^ mice were stained for their cell surface expression of CD19 and I-Aβ and analysed by FACS. Our data show high expression levels of MHC-II molecules on CD19+ cells from B6 mice compared to that from I-Aα^-/-^ mice (Figure 1a). In addition, we injected both wild type and I-Aα^-/-^ mice with gamma-ray inactivated SFV (1×10^6^ equivalent pfu/mouse) intravenously (i.v). Serum samples collected at days 3, 6, 9, 12, 15 and 21 post vaccination were tested for anti-SFV antibody responses and their isotypes were determined. The data show that antibody responses in I-Aα^-/-^ mice, in contrast to that of B6 mice, are restricted to IgM (Figure 1b). Overall, our data confirm the phenotype of I-Aα^-/-^ mice in terms of inability to antibody isotype switching and lack of MHC-II expression.

### Susceptibility of I-Aα^-/-^ mice to aSFV infections

**Figure 2:**
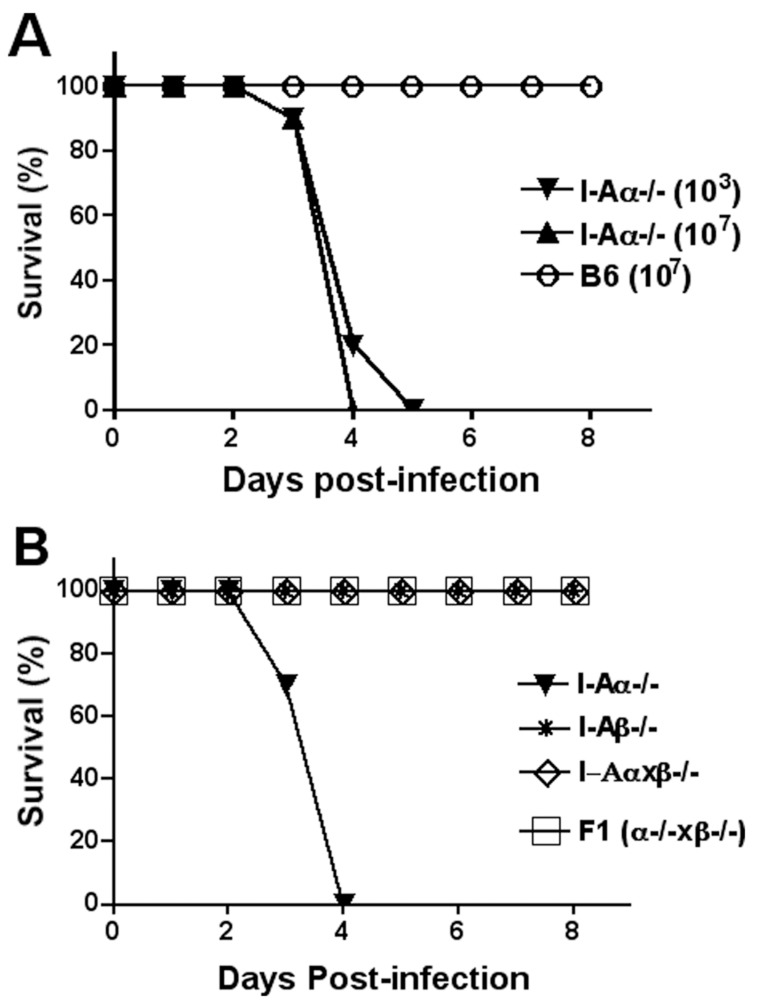
Susceptibility of I-Aα^-/-^ mice to aSFV infection. Mice were infected i.v with aSFV and animals were monitored for clinical symptoms and mortality for a period of 21 days (no changes were observed after day 8). A) Both I-Aα^-/-^ and B6 mice were compared for their susceptibility to aSFV (10^3^ or 10^7^ pfu/mouse) infection. B) Various MHC-II knockout mice (I-Aα^-/-^, I-Aβ^-/-^ and I-Aαxβ^-/-^) and F1 of (I-Aα^-/-^ x I-Aβ^-/-^) were compared for their susceptibility to aSFV (10^7^ pfu/mouse) infection.

10-week-old female mice (10 mice/group) were infected i.v with 10^3^ or 10^7^ pfu/mouse of aSFV and survival of animals was monitored for a period of 21 days. aSFV is known to cause asymptomatic infection in B6 mice [21], and consistent with this no mortality was observed (Figure 2a). In contrast, 100% mortality occurred in I-Aα^-/-^mice even with the low (10^3^ PFU) infection dose with a mean time to death of∼4 days.

We next investigated the susceptibility of I-Aβ^-/-^ mice [6] and the double knockout I-Aαxβ^-/-^ mice [8] to aSFV infection. Again, 10-week-old female mice were infected i.v with aSFV (10^7^ pfu/mouse) and survival of infected animals was monitored for 21 days. In contrast to I-Aα^-/-^mice, no mortality was observed in either I-Aβ^-/-^ or I-Aαxβ^-/-^ mice following aSFV infection (Figure 2b). In general, despite the complete deletion of MHC-II locus in I-Aαxβ^-/-^ mice, infection with aSFV was asymptomatic. Considering that I-Aαxβ^-/-^ mice lack the I-Aα chain, our data suggests that I-Aα^-/-^ mice susceptibility to aSFV infection is not a consequence of the lack of MHC-II expression and their associated function. To eliminate the possibility that I-Aα^-/-^ mice express a Mendelian dominant inheritance feature conferring susceptibility, we crossbreed I-Aα^-/-^ and I-Aβ^-/-^ mice and infected females of the F1 generation with aSFV (10^7^ pfu/mouse). As expected, F1 mice behaved like wild-type mice and no mortality was observed as a result of aSFV infection (Figure 2b).

### Susceptibility of I-Aα^-/-^ mice to virus infection is not determined by cells of haemopoietic origin

**Figure 3:**
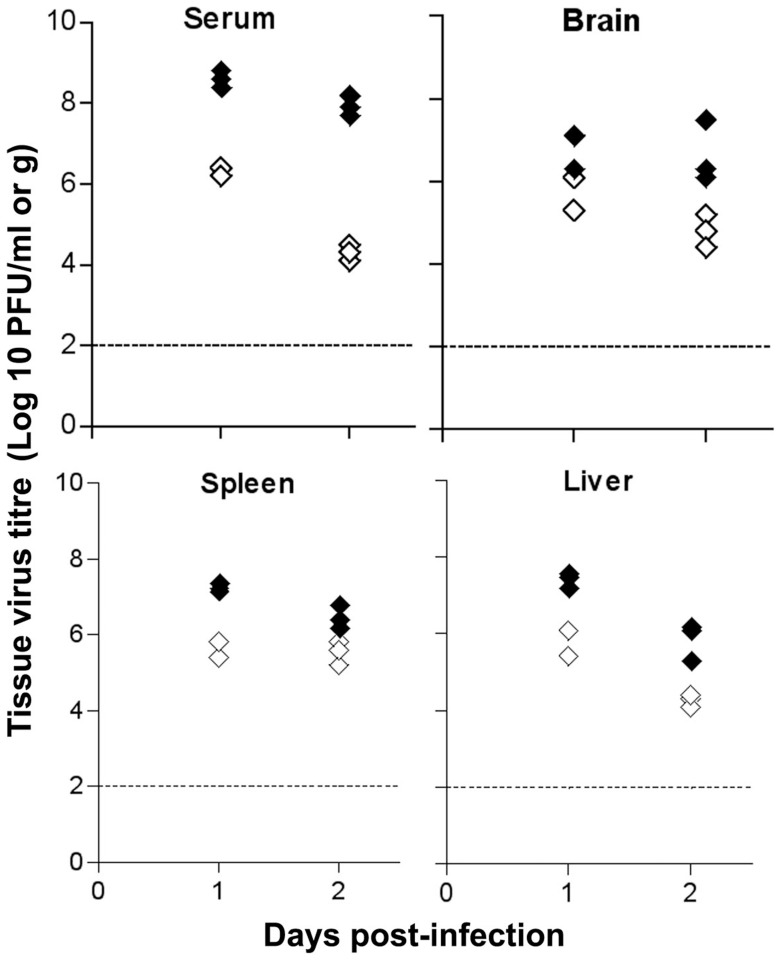
Tissue virus titres. B6 (open symbols) and I-Aα^-/-^ (solid symbols) mice were infected with aSFV (10^3^ pfu/mouse). Tissues were harvested at days 1 and 2 post infection and virus titres were estimated using plaque assay.

To understand the difference between aSFV infection in B6 and I-Aα^-/-^ mice, we determined virus titres in brain, liver, spleen and serum at days 1 and 2 post-infection. Mice were infected i.v with 10^3^ pfu of aSFV and virus titres were determined using plaque assay and expressed as pfu/g of brain, liver and spleen, or pfu/ml for serum. As shown in figure 3, I-Aα^-/-^ mice show high levels of viremia, 200 and 400 folds above titres detected in B6 mice at days 1 and 2 post-infection, respectively. This increase in viremia is associated with high virus load in all tissues tested. Statistical analysis using unpaired student *t* test show a significant difference between B6 and I-Aα^-/-^ mice in virus titres with P<0.05 for all tested time points for serum, spleen and liver samples as well as brain samples tested at day 2 post infection.

**Figure 4:**
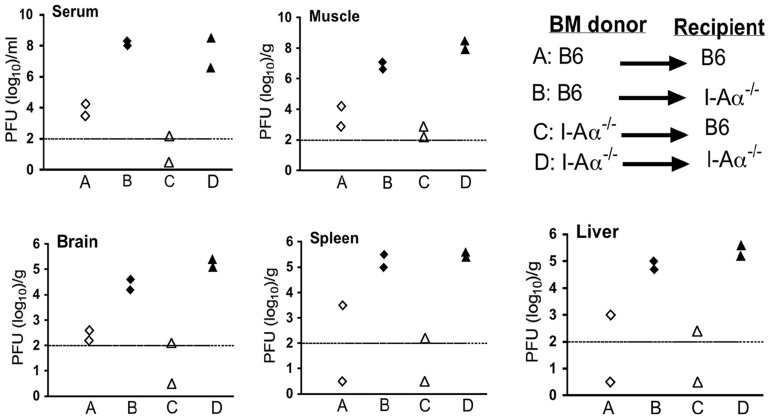
Somatic cells of I-Aα^-/-^ mice support high levels of virus replication. Females B6 and I-Aα^-/-^ mice were lethally irradiated and reconstituted with bone marrow cells from female donors of either B6 or I-Aα^-/-^ mice. Bone marrow chimeras were infected i.v with aSFV (10^3^ pfu/mouse) and tissue virus titers were estimated at day 1 post-infection using plaque assay.

Considering that MHC-II gene expression is tightly regulated and mostly restricted to cells of reticulo-endothelial origin such as thymic epithelium and APCs [1], [2] including B cells, we investigated if somatic or haematopoietic cells are involved in the susceptibility of I-Aα^-/-^ mice to aSFV infection. Females B6 and I-Aα^-/-^ mice at 6 weeks of age were irradiated with 950 Rad of gamma-rays using a ^137^Cs source at CSIRO-Canberra. Irradiated mice were reconstituted, as previously described, using 5×10^6^ bone marrow cells from female donors (either B6 or I-Aα^-/-^) instead of foetal liver [22]. Bone marrow chimeras were treated with antibiotics for 3 weeks post-reconstitution. Successful establishment of chimeric mice was confirmed by FACS analysis of cell surface expression of MHC-II on CD19^+^ cells (data not shown). 5 weeks post-reconstitution, fully chimeric mice were injected i.v with 10^3^ pfu/mouse of aSFV and tissue virus titers tested 1 day post infection using plaque assays. As shown in figure 4, various tissues from irradiated recipient I-Aα^-/-^ mice show high virus titres regardless of the genotype of the transferred bone marrow (whether B6 or I-Aα^-/-^) following aSFV infection whereas B6 recipients expressed wt phenotype irrespective of donor bone marrow origin.

### IFN-I response in I-Aα^-/-^ mice

**Figure 5:**
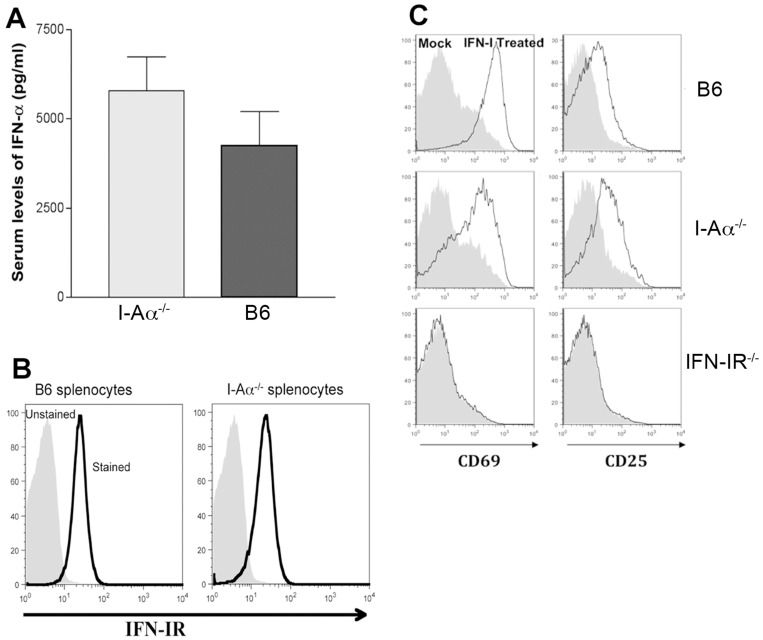
IFN-I responses in I-Aα^-/-^mice. A) I-Aα^-/-^and B6 mice were infected with aSFV and serum samples were collected 24 h post infection and tested for IFN-Iα levels. B) Splenocytes from naïve I-Aα^-/-^ and B6 mice were tested for cell surface expression of IFN-I receptor. C) Splenocytes from B6, I-Aα^-/-^ and IFN-αR1 ^-/-^ mice were incubated *in vitro* with 1000 U/ml of r-IFN-β (black line) or medium alone (filled grey) for 12 hours and tested for cell surface expression of CD69 and CD25. The expression profile of B220+ cells is shown.

The early mortality following aSFV infection in I-Aα^-/-^ mice and the increased tissue virus titres led us to investigate the possibility of a defective IFN-I response in I-Aα^-/-^ mice. Therefore, sera samples were collected from mice infected with 10^7^ pfu/mouse aSFV and tested for IFN-α levels. As shown in figure 5A, higher levels of IFN-I α were detected 24 h post aSFV infection in serum samples from I-Aα^-/-^ mice compare to B6 mice. Thus the increased viral susceptibility of I-Aα^-/-^ mice is not due to a defective IFN-I α response and the heightened IFN-I release in I-Aα^-/-^ mice is consistent with the heightened viremia in I-Aα^-/-^ mice.

We next examined the cell surface expression of IFN-I receptors and found that both wt B6 and I-Aα^-/-^ mice express similar levels (Figure 5B). We have previously reported a phenomenon of IFN-I mediated partial lymphocyte activation during acute viral infection and have illustrated the essential role of IFN-I receptors in this phenomenon [23]. To address the functionality of IFN-I receptors in I-Aα^-/-^ mice, we treated splenocytes from B6, I-Aα^-/-^, and IFN-IR^-/-^ mice with 1000 U/ml of recombinant IFN-I (r-IFN-β). Following incubation in vitro for 12 h. Cell surface expression of CD69 and CD25 was evaluated using FACS. As shown in figure 5C, splenocytes from both B6 and I-Aα^-/-^, in contrast to IFN-IR^-/-^, mice show increased expression levels of CD69 and CD25 on B220+ cells following treatment with rIFN-β. This illustrates the fact that I-Aα^-/-^ mice have functional IFN-I receptors. Importantly, IFN-I mediated anti-viral immunity involves various signalling pathways and mice defective in STAT1, for example, have been shown to be susceptible to viral infections [24]. Unlike previously published work regarding STAT1^-/-^ mice, cells of I-Aα^-/-^ mice are responsive to IFN-I treatment as evident by the induction of CD69 expression (figure 5B). Overall, our data show that the increased susceptibility of I-Aα^-/-^mice to aSFV infection is neither associated with a lack of IFN-I secretion nor a defective IFN-I receptor function. To further study this phenomenon of increased viral susceptibility, we investigated the possibility of enhanced viral replication *per se* using in vitro cultures of macrophages, splenocytes and fibroblasts.

### High level of viral replication in vitro

**Figure 6:**
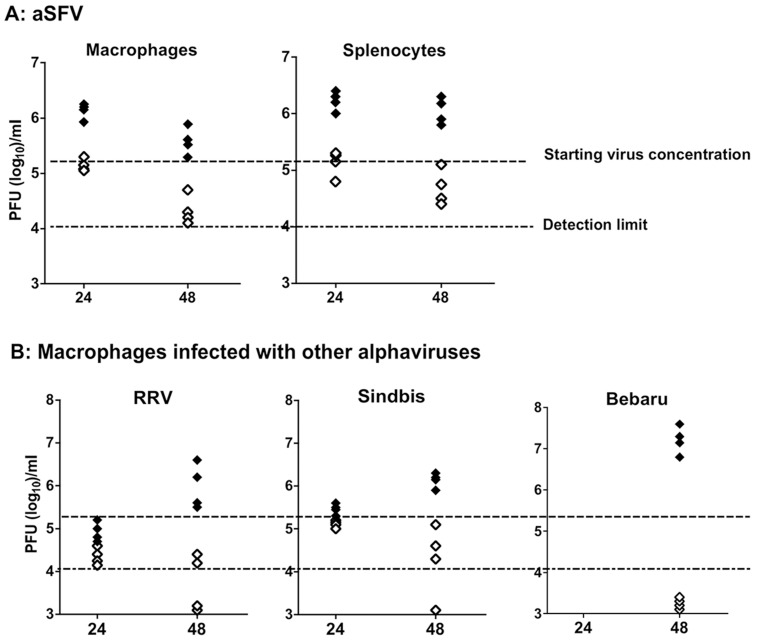
Viral replication in vitro. Splenocytes and peritoneal macrophages obtained from B6 (open symbols) and I-Aα^-/-^ (solid symbols) mice support high levels of alphaviruses replication in vitro. A) Splenocytes and macrophages were infected in vitro with aSFV using MOI of 0.5 pfu/cells. B) Macrophages were infected with RRV, Sindbis, and Bebaru using MOI of 0.5 pfu/cells. Culture supernatants were sampled 24 and 48 h post infection and virus titres were estimated using plaque assay.

B6 wt and I-Aα^-/-^ mice were injected i.p with thioglycollate (1 ml/mouse) and three days later, peritoneal macrophages and splenocytes collected. 1×10^6^ nucleated cells were infected with aSFV at a MOI of 0.5 pfu/cell. Virus replication in tissue cultures was estimated 24 and 48 hours later by plaque assay. As shown in figure 6A, significantly higher SFV replication was found in splenocyte and macrophage cultures derived from I-Aα^-/-^ mice than that from B6 mice. Residual virus titres of∼2×10^5^ after 24 h, and ∼1.5×10^4^ after 48 h were obtained following in vitro incubation of virus in the absence of cells. These control virus titres were comparable to that obtained from infected splenocyte and macrophage cultures of B6 mice, which confirms that little to no virus replication occurs in vitro in such cells of wt mice. This contrasts with high virus titres (100–200 folds higher) detected in I-Aα^-/-^ splenocyte and macrophage cultures. To test whether in vitro cultures of I-Aα^-/-^ macrophages can also support the replication of other alphaviruses, we evaluated virus replication of Ross River Virus, Sindbis and Bebaru at 24 and 48 h post-infection. As shown in figure 6B, macrophages from I-Aα^-/-^ mice, but not from B6 mice, supported significant replication of all the alphaviruses tested at 48 h post infection. These data indicate that macrophages from I-Aα^-/-^ mice, but not B6 mice, support replication of aSFV as well as other alphaviruses. It has been reported previously that in vitro aSFV infection of spleenic macrophages is associated with viral antigen presentation without release of infectious virus particles [25]. This block of viral replication/release is absent in macrophages of I-Aα^-/-^ mice.

**Figure 7:**
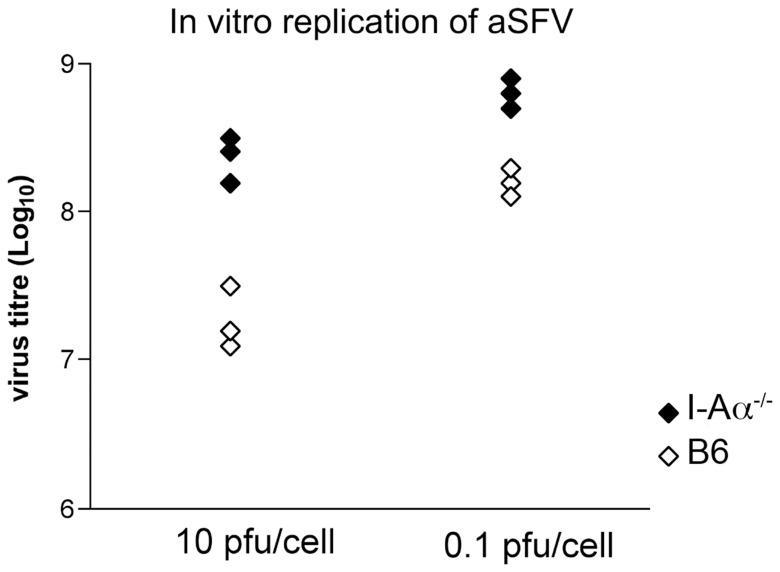
I-Aα^-/-^ cell line supports increased viral replication compared to the B6 cell line generated in parallel. 10^6^ cells from B6 (open symbols) and I-Aα^-/-^ (solid symbols) fibroblast lines were infected with aSFV (MOI of 10 or 0.1 pfu/cell) and culture supernatants were assayed 24 h later for virus titres by plaque assay.

In addition to virus replication experiments using ex-vivo cells, we generated tumor cell lines from I-Aα^-/-^and B6 mice that are not constitutively expressing MHC-II, for in vitro virus growth studies. We generated stable methyl colantherine induced I-Aα^-/-^ and B6 fibroblast cell lines using a similar protocol to that used previously for the generation of MC57 cell line from the wild type B10 mice [26]. Triplicate flasks from I-Aα^-/-^ and B6 cell lines were infected with aSFV using a MOI of 10 or 0.1 pfu/cell. Culture supernatants were harvested 24 h later and virus titres estimated using plaque assays. As shown in figure 7, significantly higher virus titres were obtained from the I-Aα^-/-^ cell line compared to that of the B6 cell line with P values of 0.018 and 0.0018 for both 10 and 0.1 pfu, respectively, using unpaired student *t* test. This data confirms that the susceptibility of I-Aα^-/-^ mice to virus infection is caused by an enhanced viral replication per se. These cell lines will be a vital tool to investigate the molecular and genetic basis of the mechanisms responsible for this unusual phenomenon.

### Susceptibility of I-Aα^-/-^ mice to other virus infections

**Figure 8:**
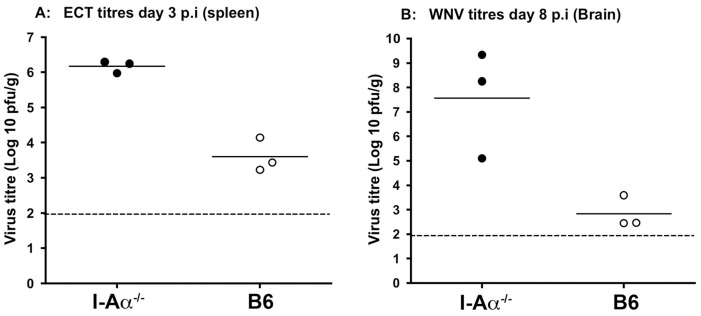
Enhanced virus replication in tissues of I-Aα^-/-^ mice. Knockout and wild-type mice were infected i.v with 10^3^ pfu/mouse of ECT-Moscow (A) or WNV (Sarafend) (B) and animals were monitored for clinical symptoms. Tissues were harvested at day 3 post infection for ECT-Moscow infected animals and day 8 post infection for WNV infected animals and virus titres were estimated using plaque assays.

To test if this increased susceptibility of I-Aα^-/-^ mice to alphavirus infections also applied to other virus infections, we tested viral replication of ectromelia (ECT) virus (poxviridae: DNA genome) and West Nile Virus (WNV) (Flaviviridae: RNA genome). I-Aα^-/-^ and wild-type mice were infected i.v with 10^3^ pfu/mouse of ECT-Moscow strain and spleens were harvested at day 3 p.i and virus titres estimated using plaque assays. The data clearly show significant enhancement of ECT replication in spleens of I-Aα^-/-^ mice compared to wild-type controls with P value of 0.001 using unpaired student *t* test (Figure 8A). In addition, all I-Aα^-/-^ mice designated to be sampled at day 6 post-infection had died prior to sampling. No-mortality occurred, as expected, in the wild-type control group. I-Aα^-/-^ mice have been used previously to investigate the role of Ab responses in the recovery from virulent ECT infection [19]. While virus replication in I-Aα^-/-^ mice was not evaluated. The researchers used s.c infection and reported that ECT-Moscow infection cause lethality (at days 12–25 post-infection) in primed and un-primed I-Aα^-/-^ mice. We suspect that the route of administration may have influenced the early mortality detected in our model (day 6 post-infection using i.v). Importantly, our data clearly illustrates the significant increase in ECT replication in I-Aα^-/-^ mice (2 log difference) compare to that of B6 mice. We also used the flavivirus WNV as an additional virus model and infected both I-Aα^-/-^ and B6 mice i.v with 10^3^ pfu/mouse and estimated brain virus titres at day 8 p.i. Our data show a significant increase in the mean virus titres (4 log difference) in brain tissues from I-Aα^-/-^ mice compared to B6 wild-type controls with P value of 0.023 using unpaired student *t* test (Figure 8B). These data indicate that I-Aα^-/-^ mice support high levels of virus replication of a variety of virus types compared to that of wt B6 mice and this is observed with viruses that employ vastly different replication strategies.

In conclusion, we have uncovered a very unusual phenomenon in I-Aα^-/-^ mice namely a vastly increased susceptibility to aSFV and other virus infections. This susceptibility is associated with enhanced virus replication (alphaviruses, flaviviruses, and orthopoxviruses) in a variety of tissues in vitro and in vivo. This increased viral susceptibility is unrelated to the MHC-II negative phenotype and appears not to involve a defect in the IFN-I mediated anti-viral response. Thus, our data indicate that I-Aα^-/-^ mice may carry two distinctive and unrelated phenotypes: a defective MHC-II expression and an enhanced virus replication not related to defective antigen presentation.

## Materials and Methods

### Ethics statement

This study was carried out in strict accordance with the recommendations in the Guide for the Care and Use of Laboratory Animals of the Australian National University. Experimental protocol was approved by the Animal Ethics Committee of the Australian National University, ACT, Australia (Permit Number: JIG47.06).

### Animals

wild type C57BL/6J (B6) mice and syngeneic mice deficient in MHC-II molecules as a result of a defective Aβ gene (I-Aβ^-/-^) [6], a disrupted Aα gene (I-Aα^-/-^) [7], a complete deletion of Aα and Aβ locus (I-Aα/β^-/-^) [8], and IFN-α receptor knockout (IFN-IR^-/-^) [27] were used in this study. Throughout this study, 10-week-old female mice were used. Mice were bred under specific pathogen-free conditions and supplied by the Animal Breeding Facilities at the John Curtin School of Medical Research, Australian National University.

### Cells and viruses

Vero (African green monkey kidney) and BHK (baby hamster kidney) were originally obtained from the *American* Type *Culture* Collection (ATCC). In addition, de novo methylcolantherine induced I-Aα^-/-^ and B6 fibroblast cell lines were prepared as has been described [26]. All cells were maintained in Eagle's minimal essential medium (EMEM) plus 5% foetal calf serum (FCS) and grown at 37°C in a humidified conditions with 5% CO_2_.

Working stocks of avirulent SFV (strain A7) were prepared by infecting semi-confluent BHK cell monolayers at a multiplicity of 0.5 pfu per cell. Infected cells were incubated for 24 h and culture supernatants harvested, centrifuged at 1200 g for 4 min to remove cell debris and stored in single-use aliquots at −80°C. Virus titre determined by plaque assay on Vero cells and the titer of the working stocks of aSFV was 1×10^8^ pfu/mL. Other alphaviruses (Ross River virus, Sindbis virus, and Bebaru), ectromelia virus (orthopoxvirus) and West Nile Virus (Flavivirus), from previously published work [28]–[30], have also been used in few experiments. For inactivated SFV, virus stocks were subject to 1.3×10^6^ Rad of gamma-irradiation (137Cs source at CSIRO-Canberra) and virus sterility was confirmed by lack of plaque formation on vero cells.

### Plaque assay

Virus titres were estimated by plaque formation on semi-confluent monolayers of Vero cells as previously described [31]. Briefly, samples were serially diluted on ice in HBSS, pH 7.6, containing 0.2% BSA, and cells inoculated in duplicate with 0.1 ml aliquots of the diluted samples. Adsorption was for 1 h at 37°C followed by the addition of an agar overlay medium. After 48 h incubation at 37°C, cells were fixed with 5% paraformaldehyde (BDH chemicals, Aust.) for 1 h. Over-layers were removed, and fixed cells were stained with 0.2% crystal violet in ddH_2_O. The stain was aspirated and plaques counted.

For virus determination in infected mouse tissues, animals were sacrificed at a given time post-infection, tissues were aseptically removed, snap-frozen in liquid nitrogen or dry ice, and stored at −70°C. 10% (weight/volume) tissue suspensions in ice-cold HBSS (pH 7.6) containing 0.2% BSA were homogenized, clarified by centrifugation (18,000×g for 5 min at 4°C), and supernatants were stored in aliquots at −80°C. The limit of virus detection by plaque assay in tissues and serum samples of infected mice was 10^2^ pfu/g or ml, respectively. In addition, brain, spleen, and muscle tissues from uninfected animals were homogenised in the presence of a known amount of virus and used as controls. Plaque titration indicated no loss of virus titers due to tissue components released during homogenisation. Virus determination for ECTV and WNV was carried out as previously described [29], [30].

### IFN-α levels in sera

Sera from SFV-infected B6 and I-Aα^-/-^ mice were collected at day 1 p.i. (3 mice/strain) and tested for IFN-α levels using a sandwich ELISA kit according to the manufacturer's instructions (US Biological). For each experiment, a standard curve in the range of 0 −500 pg/ml IFN-α was generated and used to estimate the concentration of serum IFN-α of tested samples. Detection limit for IFN-α was 12.5 pg/ml.

### Flow cytometric analysis

Spleens from infected and control mice were harvested and red cell-depleted single cell suspensions prepared. Lymphocytes (1×10^6^) were stained using fluorescent-conjugated anti-CD3, -CD4, -CD8, -CD19, -B220, -CD69, and -CD86 specific Abs (BD Pharmingen) and cell surface expression of these markers was assessed by flow cytometry using BD LSR II (BD Biosciences, Australia). In addition, cell surface expression levels of IFN-I receptor on splenocytes from B6 and I-Aα^-/-^ mice were assessed by flow cytometry using BD LSR II (BD Biosciences, Australia) using I-Aβ specific Abs (BD Pharmingen). Dead cells were labeled with 7-aminoactinomycin D (Sigma-Aldrich) and Fc receptors were blocked by the addition of anti-mouse CD16/CD32 (Fc III/II receptor) Ab (BD Pharmingen). Fc receptor Ab and 7-aminoactinomycin D were added before the addition of cell subpopulation- and activation marker-reactive Abs.

### Bone Marrow Chimeras

Females B6 and I-Aα^-/-^ mice were lethally irradiated with 950 Rad from a ^137^Cs source (CSIRO-Canberra) at 6 weeks of age, and reconstituted, as previously described [32], with 5×10^6^ bone marrow cells. Bone marrow chimeras were treated with antibiotics (Neomycin and Polymyxin B sulfate) for 3 weeks post-reconstitution. 2 weeks later (5 weeks post-reconstitution), 2 mice from each group were sacrificed and splenocytes were stained using fluorescent-conjugated anti-CD19 and I-Aβ specific Abs (BD Pharmingen) and cell surface expression of MHC-II on CD19^+^ cells was assessed using flow cytometry using BD LSR II (BD Biosciences, Australia). The remaining mice were injected i.v with 10^3^ pfu/mouse of aSFV and various tissues were harvested at predetermined time points to test for virus titers using plaque assay.

### Statistical analysis

Data were expressed as mean ± standard error of means (SEM) and analysed using a two-tailed student *t* test. A *p*-value of<0.05 was deemed significant.
